# A novel graph-based k-partitioning approach improves the detection of gene-gene correlations by single-cell RNA sequencing

**DOI:** 10.1186/s12864-021-08235-4

**Published:** 2022-01-07

**Authors:** Heng Xu, Ying Hu, Xinyu Zhang, Bradley E. Aouizerat, Chunhua Yan, Ke Xu

**Affiliations:** 1 Department of Psychiatry, Perelman School of Medicine, University of Pennsylvania, Philadelphia, PA USA; 2 Center for Biomedical Information and Information Technology, National Cancer Institute, Rockville, MD USA; 3 Department of Psychiatry, Yale School of Medicine, New Haven, CT USA; 4 Connecticut Veteran Healthcare System, West Haven, CT USA; 5 Bluestone Center for Clinical Research, College of Dentistry, New York University, New York, NY USA

## Abstract

**Background:**

Gene expression is regulated by transcription factors, cofactors, and epigenetic mechanisms. Coexpressed genes indicate similar functional categories and gene networks. Detecting gene-gene coexpression is important for understanding the underlying mechanisms of cellular function and human diseases. A common practice of identifying coexpressed genes is to test the correlation of expression in a set of genes. In single-cell RNA-seq data, an important challenge is the abundance of zero values, so-called “dropout”, which results in biased estimation of gene-gene correlations for downstream analyses. In recent years, efforts have been made to recover coexpressed genes in scRNA-seq data. Here, our goal is to detect coexpressed gene pairs to reduce the “dropout” effect in scRNA-seq data using a novel graph-based k-partitioning method by merging transcriptomically similar cells.

**Results:**

We observed that the number of zero values was reduced among the merged transcriptomically similar cell clusters. Motivated by this observation, we leveraged a graph-based algorithm and develop an R package, scCorr, to recover the missing gene-gene correlation in scRNA-seq data that enables the reliable acquisition of cluster-based gene-gene correlations in three independent scRNA-seq datasets. The graphically partitioned cell clusters did not change the local cell community. For example, in scRNA-seq data from peripheral blood mononuclear cells (PBMCs), the gene-gene correlation estimated by scCorr outperformed the correlation estimated by the nonclustering method. Among 85 correlated gene pairs in a set of 100 clusters, scCorr detected 71 gene pairs, while the nonclustering method detected only 4 pairs of a dataset from PBMCs. The performance of scCorr was comparable to those of three previously published methods. As an example of downstream analysis using scCorr, we show that scCorr accurately identified a known cell type (i.e., CD4+ T cells) in PBMCs with a receiver operating characteristic area under the curve of 0.96.

**Conclusions:**

Our results demonstrate that scCorr is a robust and reliable graph-based method for identifying correlated gene pairs, which is fundamental to network construction, gene-gene interaction, and cellular omic analyses. scCorr can be quickly and easily implemented to minimize zero values in scRNA-seq analysis and is freely available at https://github.com/CBIIT-CGBB/scCorr.

**Supplementary Information:**

The online version contains supplementary material available at 10.1186/s12864-021-08235-4.

## Background

Single-cell RNA sequencing (scRNA-seq) enables transcriptome profiling at high cell resolution and provides unprecedented precision in identifying the molecular mechanisms underlying disease [1, 2]. Advances in scRNA-seq have promise for uncovering novel or rare cell types [2, 3], tracking the trajectories of cell lineages during cell development [4], and identifying cell type-specific genes for diseases [5, 6] and for cancer treatment responses [7]. However, computational scRNA-seq analysis remains challenging, which limits the applications of scRNA-seq for biological discovery.

A central challenge to cell type identification and downstream analysis is the abundance of zero values, known as “dropout”, in single cells due to either low transcript copy number [8] and/or ineffective capture capacity of scRNA-seq technology [9, 10]. scRNA-seq typically captures only 5–15% of the transcriptome of each cell [11]. Dropout causes significant zero inflation, increasing background noise, which leads to loss of detection of gene-gene correlations crucial to gene network construction and determination of lineage relationships among cells.

Considerable computational effort has been expended to address scRNA-seq dropout [12–17]. One approach is to aggregate cells using small proportions of highly variable genes that are heavily weighted in analysis [18]. Another approach is to impute zero values [13, 14, 19]. For example, DeepImpute employs a deep neural network imputation algorithm that uses dropout layers to identify patterns in scRNA-seq data to impute zero values [12]. Markov affinity-based graph imputation of cells imputes likely missing expression data to detect the underlying biological structure via data diffusion [20]. A newly developed algorithm embraces zero values based on binary zero/nonzero patterns into the analysis to improve gene-gene correlation and gene network analysis [21]. Leveraging bulk RNA-seq data as a constraint, the SCRABBLE method reduces the bias toward expressed genes in the imputation process and enables the capture of cell-cell and gene-gene relations [22]. A network-based imputation model has been recently proposed to handle noisy data and to improve cell type identification [23]. While these methods reduce dropout, recovering gene-gene relationships from zero abundant data remains challenging due to the noise introduced by imputation of a large number of zero values or loss of information by simplifying the complexity of data.

A common practice in examining gene-gene correlations is estimating the correlation coefficient among gene pairs using Pearson, Spearman, or Cosin analysis. Excessive zero values in scRNA-seq data result in a biased estimation for gene-gene correlation. Several methods have been reported to recover coexpressed genes to reduce dropout noise. The scImpute method allows us to simultaneously determine which values are affected by dropout while imputing zero values only on dropout entries [24]. This method estimates a dropout probability in each cell and imputes the high probable dropout values in a cell by referring to information of the same gene in transcriptomically similar cells. Bageritz et al. selected genes with only a number of unique molecular identifiers greater than 2000 to perform gene-gene correlation [25]. This method excluded cells contributing to the strongest gene-gene correlation coefficient to avoid outlier bias. One limitation of the filtering method is that it may filter out biologically expressed genes with low expression. In addition to using Pearson correlation, permutation-based estimation is also reported to identify coexpressed gene pairs such as correlatePair [26]. These methods have been applied to different datasets for gene-gene expression analysis. Nevertheless, little effort has been devoted to accurately identifying coexpressed genes. More tools are needed to address this challenge.

Here, we present “scCorr” (single-cell gene-gene correlation), a novel graph-based k-partitioning approach to address dropout and to recover missing gene-gene correlations. The motivation of scCorr is based on an observation that zero values are markedly reduced by merging cells with similar transcriptome profiles. Our goal is to limit dropout effects and to recover gene-gene correlations without imputing zero values. Specifically, the scCorr algorithm includes 1) generating a graph or topological structure of cells in scRNA-seq data; 2) partitioning the graph into k multiple min-clusters employing the Louvain algorithm, with cells in each cluster being approximately homologous (with similar transcriptional profiles); 3) visualizing the series of k-partition results to determine the number of clusters; 4) averaging the expression values, including zero values, for each gene within a cluster; and 5) estimating gene-gene correlations within a partitioned cluster.

In this study, we demonstrate that the graph k-partitioning approach enables the reliable acquisition of cluster-based gene-gene correlations in two independent peripheral blood mononuclear cell (PMBC) scRNA-seq datasets: one from healthy participants (dataset 1) and another dataset including wild-type control cells and knockout cells from a patient with T cell deficiency (dataset 2). In addition, we applied scCorr in a scRNA-seq dataset from relatively homogenous cells derived from the central nervous system (dataset 3). We applied scCorr to estimate gene-gene correlations in these datasets and compared the performance of gene pair identification between scCorr and the nonclustering single-cell gene correlation method in each dataset. To validate the scCorr method, we compare the performance of scCorr and three published methods mentioned above: scImpute, filtering method, and correlatePair. Finally, we show that scCorr can accurately identify a known cell type (i.e., CD4+ T cell) from a PBMC scRNA-seq dataset.

## Results

### Reduction of zero value abundance in merged cells

**Fig. 1:**
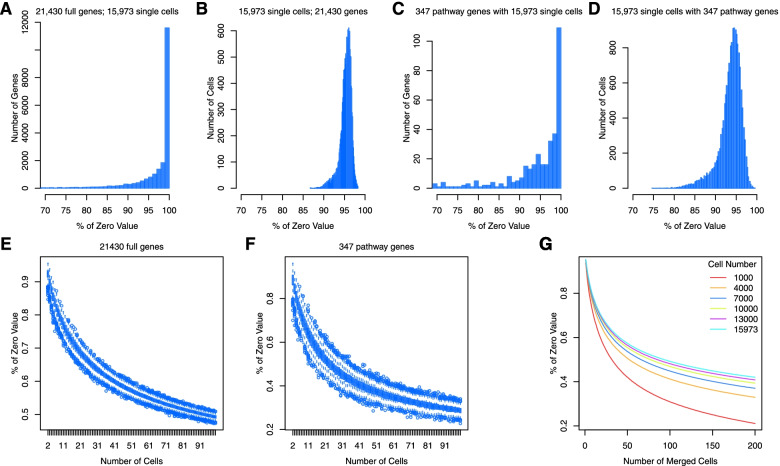
Study principles and workflow. Data are from single-cell RNA-seq of peripheral blood monocyte cells in healthy participants from dataset 1. **A**-**D** present the distribution of zero values by individual genes and by single cells. A total of 21,42830 genes had zero values in at least one cell (**A**), and more than 95% of 15,973 cells showed zero values in at least one gene  (**B**). Among a set of 347 genes from 3 KEGG pathways, all genes had zero values in at least one cell (**C**), and 95% of 15,973 cells contained zero values in at least one gene (**D**). **E**-**G** show reductions of zero values in merged cells. The percentage of zero values of 21,430 genes was markedly reduced in merged cells. The zero-value reduction was approximately 50% among 50 merged cells (**E**). Similarly, zero values of the 347 genes were reduced in merged cells (**F**) and were consistently observed in 6 different cell sets (**G**)

A total of 21,430 genes were annotated from 15,973 PBMCs from two healthy subjects in dataset 1 [27]; we observed that 21,428 out of the 21,430 genes had zero values in a cell (Fig. 1A), and 95% of the 15,973 cells showed at least one undetected gene with zero values (Fig. 1B), suggesting that scRNA-seq captures only 5% of gene expression at the single-cell level. For example, among the 347 genes in 1532 biologically related gene pairs on 3 Kyoto Encyclopedia of Genes and Genomes (KEGG) pathways (i.e., hsa04010, hsa04115, and hsa04662) (Table S1), over 70% exhibited zero expression (Fig. 1C, D), resulting in poor gene-gene correlation among these known gene pairs. These data underscore the importance of developing a method to minimize zero value inflation to recover gene-gene correlations.

Interestingly, we observed that merging cells with similar transcriptomic profiles dramatically reduced the proportion of zero values. In a set of 50 merged cells that were randomly selected from the 15,973 PBMCs, the percentage of zero values from the same set of 21,430 genes was reduced from 90% in two cells to 57.4% (95% confidence interval [CI), 57.3, 57.4%] in the 50 merged cells (Fig. 1E). Revisiting the same 347 genes from the 3 KEGG pathways, the reduction in zero values increased further, from 90% in two cells to 37.6% (95% CI: 37.5, 37.8%) in the same 50 merged cells (Fig. 1F). Examining the impact of the number of merged cells on zero value frequency, focusing on six cell populations of 1000 to 15,973 cells, the reduction of zero value proportion stabilized when the merged cells exceeded 100 in a set of 1000 cells (Fig. 1G). Simulation analysis of four separate sets of cells and genes further supported the reduction of zero values in merged cells (Supplementary Fig. 1A and B). This property motivated the development of the scCorr package to improve the detection of gene-gene relationships by clustering cells with similar profiles.

### Graph-based cell clustering

**Fig. 2:**
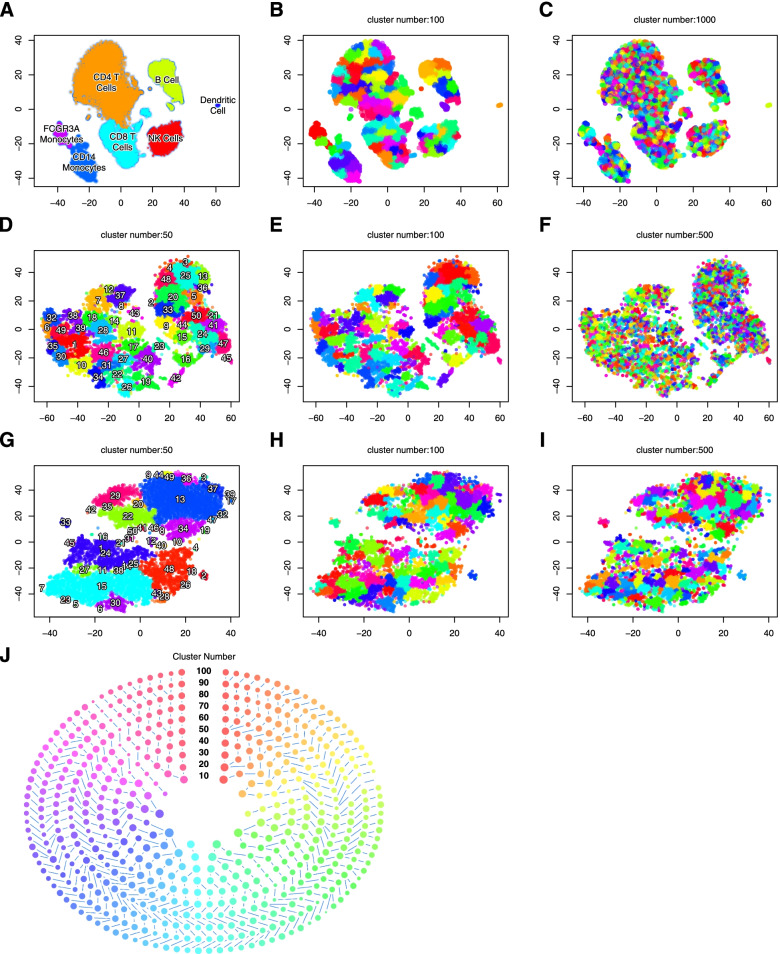
Workflow and features of the scCorr method. Data dimensional reduction and cell classification by tSNE and cell type identification using the marker gene approach in dataset 1 (**A**). Cell partitioning is based on a tSNE plot using scCorr with different numbers of clusters in dataset 1 (**B**: k = 100; **C**: k = 1000). scCorr partitioned different numbers of clusters for wild-type cells in dataset 2 (**D**: k = 50; **E**: k = 100; **F**: k = 500) and for knockout cells in dataset 2 (**G**: k = 50; **H**: k = 100; **I**: k = 500). **J**: tracing of the evolution of each partitioned cluster

scCorr is based on local optimal modularity (i.e., the Louvain algorithm) to partition a graph into k clusters. Louvain, an unsupervised algorithm, includes greedy optimization and community aggregation steps [28]. Figure 2 presents the analysis strategy of scCorr on the scRNA-seq data of 21,430 genes in 15,973 cells from the healthy PBMC samples, dataset 1. Figure 2A shows a conventional tSNE plot of the cell clusters and cell type identification. Using our scCorr package, the graph edges were weighted by the distances and distance matrix, which was converted to a weighted graph determined by a cutoff of three as the default (any edge weight > 3 is removed from the graph), and an initial number of N clusters was generated with N greater than k. Next, the center of each cluster was calculated, and adjacent clusters with the smallest distance were merged one by one until N equaled k. As presented in Fig. 2B (k = 100) and 2C (k = 1000), scCorr-partitioned clusters did not change the local structure of transcriptomically defined cell populations. A series partitioning process was carried out to determine the desired number of clusters (Supplementary Fig. 2).

Similarly, we applied scCorr to partition cell clusters in the second dataset, which contains 8189 wild-type cells from healthy subjects and 8334 knockout cells from a patient with inherited human T cell deficiency [29]. tSNE plots show different cell types between wild-type and knockout cells. Figure 2D-F presents scCorr partitioned into different numbers of cell clusters (k = 50 ~ 500) in wild-type cells, and Fig. 2G-I presents scCorr partitioned cell clusters in knockout cells (k = 50 ~ 500). One of the utilities included in the scCorr package is able to trace how a cluster evolves during k graph partitioning in multiple cluster sets, displaying ladder and circle plots to visualize the evolutionary process of each cell cluster in a variety of cluster sizes (Supplementary Fig. 3). As an example, Fig. 2J depicts the evolutionary process for each cluster during graph partitioning.

**Fig. 3:**
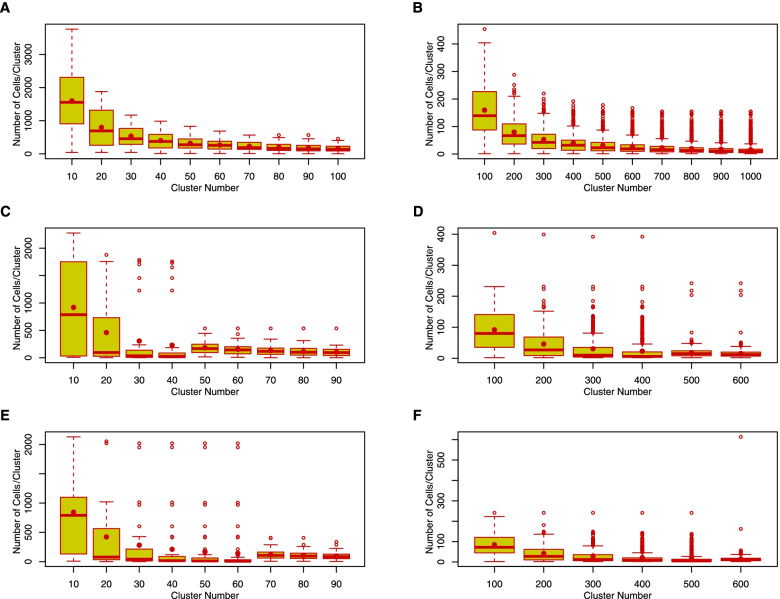
Distribution of cell numbers per cluster in different sets of cluster sizes. The red dot is the expected number of cells in a cluster, estimated by the number of cells/number of clusters in a given set of clusters. The red line is the observed median number of cells in a given set of clusters, which is close to the expected value. **A** and **B**: Healthy PBMCs from dataset 1. **C** and **D**: Wild-type T cells from dataset 2. **E** and **F**: Knockout T cells from dataset 2

We next evaluated the number of cells contained in a set of scCorr partitioned clusters. Figure 3 displays cell number distributions for each cluster set in dataset 1 (Fig. 3A and B) and dataset 2 (Fig. 3C and D, wild type; Fig. 3E and F, knockout cells). For each box plot, the observed number of cells for each cluster (median) was close to an expected number of cells per cluster, which was estimated by the number of cells/number of clusters in a given set of clusters. As shown in Fig. 3, the observed number of cells in a set of clusters generated by scCorr was similar to the expected number of cells in a set of clusters. There were only a few outliners with too many or too few cells in each cluster. For example, among a set of 100 clusters in two datasets, we observed that only 1 cluster was exceedingly large across the three panels of Fig. 3B, D, and F, suggesting that scCorr partitions cell clusters in an unbiased manner. For those clusters with fewer than 10 cells/cluster, we recommend removing or combining the clusters with other cluster sets. The rationale is that a proportion of zero values can be reduced from 95 to 75% by merging 9 cells, as shown in Fig. 1E. Thus, the cluster number in an analysis can be determined based on a total number of cells and a desired number of cells per cluster.

### Gene-gene correlation by scCorr

**Fig. 4:**
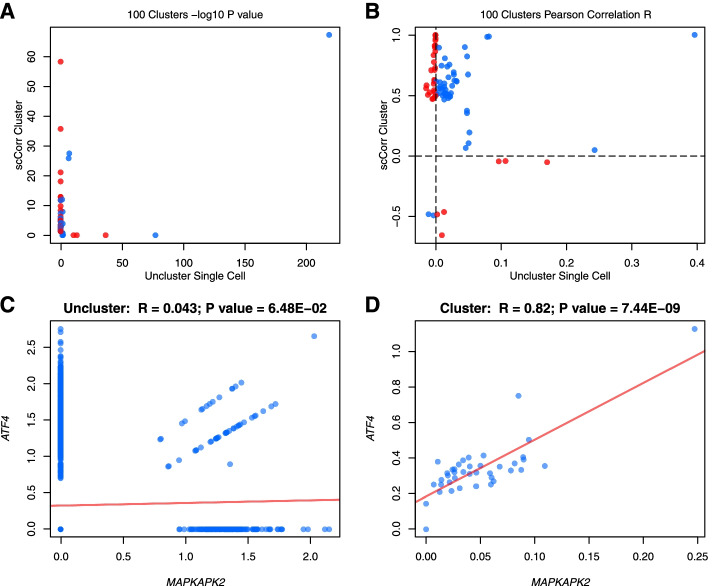
Evaluations of scCorr performance in gene-gene correlations among CD4+ T cells in dataset 1. Gene-gene relationships were quantified by Pearson correlation. In **A** and **B**, gene-gene correlation was separately performed in nonclustering single cells (X-axis) and in scCorr-partitioned cell clusters (K = 100) (Y-axis). Only the top 100 significantly correlated gene pairs are shown. Blue dots indicate agreement, and red dots indicate opposite directions of gene-gene correlation between the two methods. Correlated genes are shown as –log_10_ P- (**A**) and R- (**B**) values. scCorr detected a significant correlation between *MAPKAPK2* and *ATF4* genes (**C**), while the conventional noncluster single-cell-based method showed no significant correlation between the two genes (**D**). Gene-gene correlation varied in different numbers of clusters

We estimated gene-gene correlations in the CD4^+^ T cell population in two datasets separately. scCorr estimates the gene-gene correlation coefficient using Pearson correlation analysis by averaging the expression level of a gene within a k-partitioned cluster. Conventional nonclustering single-cell-based methods estimate gene correlation simply using Pearson correlation in the expression of a gene in individuals without considering dropout. To validate the efficiency of scCorr, we focused on the same 347 genes and 1532 gene pairs defined by the 3 KEGG pathways. In dataset 1, we present the results of scCorr-partitioned cluster sizes from 40 to 1000, which shows greater gene-gene correlation coefficients and identifies more gene pairs than the conventional nonclustering single-cell-based method. For example, in a set of 100 clusters, scCorr and the nonclustering method identified a combined total of 85 of 11,242 possible gene pairs (71 solely by scCorr, 10 solely by the nonclustering single-cell method), with only 4 detected by both methods [false discovery rate (FDR) < 0.05]. Gene-gene correlations were stronger among the pairs detected by scCorr than by the nonclustering single-cell method (62 of 85 [73%] of the gene pairs with r > 0.5 were detected by scCorr, while 4 of 85 [5%] had r > 0.1 using the nonclustering single-cell-based method). Among significant gene pairs detected by both methods, approximately 50% of gene-gene correlations were in agreement (blue dot), and the other 50% were in disagreement (red dot) in the direction of the correlations (Fig. 4). As an example, Fig. 4A and B presents the p and r values of the top 45 gene pairs selected by *p* value (scCorr: 40, nonclustering single cell-based method: 14, overlapping: 9). For example, *ATF4* and *MAPKAPK2* are a well-established pair of coexpressed genes. The nonclustering gene-gene correlation method showed no significant correlation between the two genes (*r* = 0.043; *p* = 6.48E-02) (Fig. 4C). However, scCorr significantly increased the coexpression of *ATF4* and *MAPKAPK2* (*r* = 0.82; *p* = 7.44E-09) (Fig. 4D). Similarly, scCorr outperformed the correlation of another coexpressed gene pair, *MAPK1 and DUSP2* (scCorr: *r* = 0.586, noncluster single cell-based method: *r* = 0.007) (Supplementary Fig. 4). Of note, *scCorr detected no significant correlations for randomly selected genes*. These results suggest that scCorr shows robust detection of correlated gene pairs by minimizing zero-value effects in merged cell clusters.

**Fig. 5:**
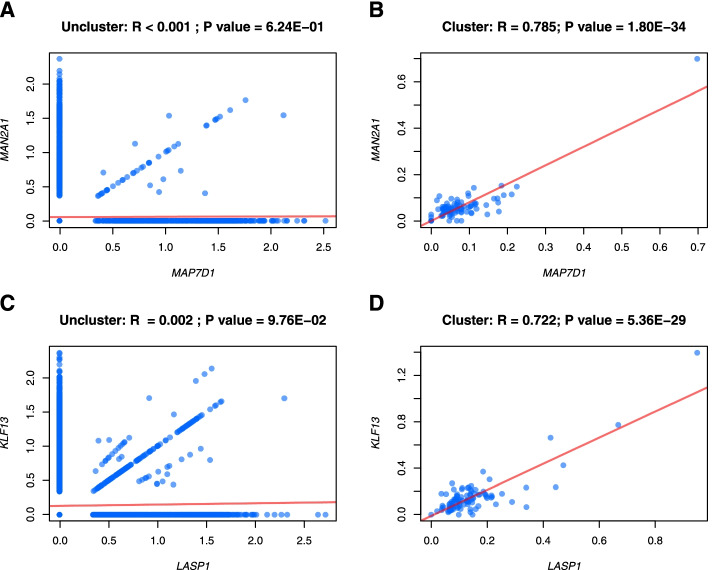
Evaluations of scCorr performance in gene-gene correlations in wild-type and knockout cells in dataset 2. In wild-type cells, the nonclustering single-cell correlation method showed no significant correlation between *MAP7D1* and *MAN2A1* (r < 0.001; *p* = 0.624) (**A**), while cluster-based scCorr showed a significant correlation between the two genes (*r* = 0.785; *p* = 1.80E-34) (**B**). In knockout cells, the nonclustering single-cell correlation method showed no significant correlation between *LASP1* and *KLF13* (r < 0.001; *p* = 0.100) (**C**), while cluster-based scCorr showed a significant correlation between the two genes (***r*** = 0.722; *p* = 5.36E-29) (**D**)

Consistent with the findings from dataset 1, scCorr identified more significantly correlated gene pairs in both wild-type and knockout cells than the nonclustering method in dataset 2. For instance, in wild-type T cells, we found no significant correlation in expression between *MAP7D1* and *MAN2A1* using the nonclustering single-cell method (r < 0.001, *p* = 0.624) (Fig. 5A). However, the expression of these two genes was highly correlated by scCorr (*r* = 0.785, *p* = 1.80E-34) (Fig. 5B). In knockout T cells, a nonclustering single-cell-based method revealed no significant correlation between the *LASP1* and *KLF13* genes (*r* = 0.002; *p* = 0.100) (Fig. 5C). scCorr detected a significant correlation of this gene pair (*r* = 0.722, *p* = 5.36E-29) (Fig. 5D).

### Validation of performance by scCorr in an additional dataset from the human central nervous system

In addition to two PBMC scRNA-seq datasets, which are highly heterogeneous, we partitioned cell clusters in scRNA-seq from relatively homogenous T cells derived from the human central nervous system, dataset 3 [30]. After quality control (QC), the dataset contained 37,377 cells and 19,711 genes. scCorr partitioned 800 mini clusters in the data (Supplementary Fig. 5). The average number of cells in each cluster was 374 across 100 clusters. We compared the performance of gene-gene coexpression between scCorr and the conventional nonclustering single-cell method. We focused on the same gene sets from three KEGG pathways as we described earlier (i.e., hsa04010, hsa04115, hsa04662). A total of 360 genes and 1781 possible pairs were mapped to three pathways in this dataset (Table S2). scCorr identified 626 significant pairs of correlated genes, while the nonclustering single-cell method identified 612 significant pairs of correlated genes. The correlation coefficients of paired genes by scCorr were greater than those of gene pairs by the nonclustering method (Supplementary Fig. 6). These results show that scCorr is a robust tool to identify coexpressed genes in homogenous cell types and can be generalized to other scRNA-seq beyond PBMCs.

### Comparisons of scCorr with three published methods

**Fig. 6:**
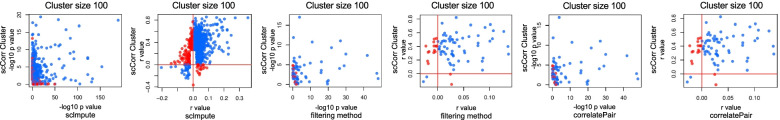
Gene-gene correlation detected by scCorr and three published methods in a single-cell RNA-seq dataset 3 (Transcriptomic and clonal characterization of T cells in the human central nervous system): scImpute, filtering method, and correlatePair. Blue dots indicate agreement, and red dots indicate opposite directions of gene-gene correlation between the compared two methods

We compared the performance of gene-gene correlation analysis between scCorr and three other methods: scImpute [24], filtering method [25], and correlatePair [26] in the same scRNA-seq dataset [30] (Fig. 6). We performed gene-gene correlation analysis on three KEGG pathways as we described, which contained a total of 360 unique genes and 1781 gene pairs. The total number of gene pairs and significant gene pairs varied among the four methods (scCorr, scImpute, filtering method, and correlatePair). scCorr identified 1769 gene pairs, and 35% of them were significant. scImpute identified 30% significant pairs among 1781 gene pairs. The filtering method identified 32% significant pairs in a set of only 152 gene pairs, missing the majority of gene pairs. The CorrelatePair method identified only 9% of significant pairs in a set of 1757 gene pairs. Thus, scCorr recovered more significant gene pairs than the three methods.

### Evaluation the efficiency of scCorr

**Fig. 7:**
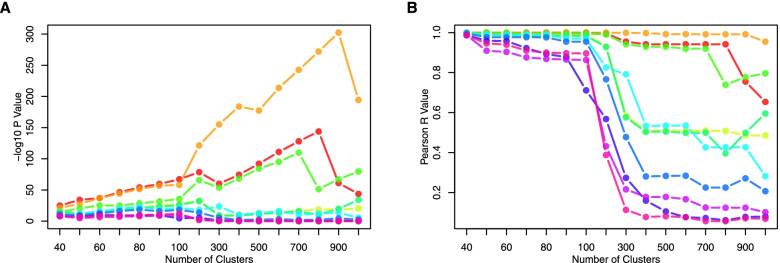
Top 10 correlated genes in different cluster sizes partitioned by scCorr among CD4+ T cells in dataset 1 evaluated by –log_10_ p- (**A**) and rr- (**B**) values

One key step in scCorr is to establish a cutoff for a reasonable cluster size. We recommend performing a series of correlation analyses with different cluster sizes to identify the cluster size at which stable *p* and r values in the cluster sets are achieved (Supplementary Fig. 7). For example, in a set of the top 10 most significant gene pairs in cluster sizes from 40 to 1000 in CD4^+^ T cells from dataset 1, the -log10 *p* and r values were relatively stable in cluster sizes from 40 to 100 (Fig. 7A and B). However, gene-gene correlations changed dramatically when the cluster size exceeded 100. As a result, a size of 100 clusters containing 125 cells per cluster showed the greatest correlation coefficients and smallest *p* values in dataset 1, indicating that a cluster size of 100 is a reasonable cutoff to achieve consistent gene-gene correlations.

scCorr requires a reasonable amount of computational time. Users are able to adjust the computational time by estimating a range of scales and k partitioning clusters (Supplementary Fig. 8A). In a set of 5967 cells, the estimated computation time for a scale of 200 is approximately 10 min (Supplementary Fig. 8B). The computation time for a scale of 400 is estimated to be 20 min for a set of 15,973 cells (Supplementary Fig. 8C). The efficiency of scCorr expands the applications of the package.

### Applying scCorr for cell type identification

**Fig. 8:**
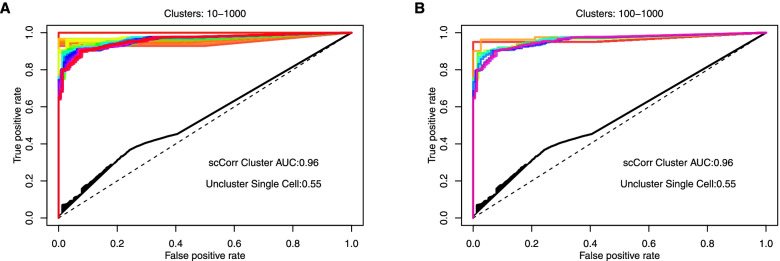
Identification of CD4+ T cells from peripheral blood mononuclear cells using single-cell RNA-sequencing data by the receiver operating characteristic area under the curve (AUC) in dataset 1. Both of the average AUCs by the scCorr method for both k = 10–1000 clusters (**A**) and for k = 100–1000 clusters (**B**) were 0.96; the AUC for the nonclustering single-cell method was 0.55

Finally, we tested the efficiency of scCorr in predicting cell type classification. We performed 10-fold cross-validation to predict CD4^+^ T cells in dataset 1. We tested the accuracy of cell type identification by scCorr in a variety of cluster sizes. The receiver operating characteristic area under the curve (AUC) was used to evaluate the prediction accuracy. Figure 8A shows the AUC of the 100 classification results of cell type identifications using scCorr partitioned clusters from 10 clusters to 1000 clusters incremental by 10 clusters. Similarly, Fig. 8B shows the AUC of 10 classification results using scCorr partitioned clusters from 100 to 1000 clusters incremental by 100 clusters. The average AUC was 0.96 across the cell type identification tests, suggesting that scCorr accurately predicted CD4+ T cells. In contrast, the performance of nonclustering single-cell-based prediction in the same numbers of nonclustering single cells was poor (i.e., AUC = 0.55), suggesting that scCorr-based cell type identification is reliable and accurate and outperforms the noncluster single-cell-based approach.

## Discussion

Built on the observation that merging cells can decrease the number of zero values in each given cell type, scCorr is developed based on a graphic structure in cells with similar transcriptomic profiles to estimate gene expression values, including zero values, in a local cell community. Unlike imputation methods, which infer missing gene expression, possibly introducing noise into the data, scCorr maintains local community data organization in single cells by k-partitioning a graph into mini-clusters in a similar cell-cell matrix based on transcriptomic similarities within the cell type. Thus, the merged cells in a partitioned cluster increase the power to recover correlated gene pairs.

Leveraging two independent scRNA-seq datasets, we show that cluster-based analysis by scCorr k-partitioning can detect more significant gene pairs than nonclustering single-cell-based analysis, suggesting that scCorr is a robust approach to address gene expression correlations in dropout scRNA-seq. scCorr is simple and fast in the R environment. We have shown that scCorr outperforms nonclustering gene-gene correlation in individual cells from a relatively homogenous dataset. The difference sets of detected significant gene pairs between scCorr and the nonclustering correlation method were greater in PBMCs than in T cells only, suggesting that scCorr may have a greater advantage in highly heterogeneous cells than in relatively homogenous datasets. However, more datasets are needed to draw a conclusion. These findings suggest that scCorr can be broadly applied to single-cell genomic analysis and is particularly useful for downstream analyses, such as differential gene expression analysis, gene-set enrichment analysis, and network construction at single-cell resolution.

Comparing robustness and efficacy among different methods is challenging due to the lack of a gold standard. In the same set of genes on three KEGG pathways, scCorr identified a greater number of correlated gene pairs than the other three published methods. The numbers of significant pairs detected by scCorr and scImpute were close to each other (612 versus 544 pairs). Both methods incorporate local information of a gene in cells with similar transcription profiles. The filtering method detected a smaller number of gene pairs (50 pairs), partially because the filtering method considers only highly expressed genes in the dataset. The correlatePair method also includes a step of filtering out low copy expressed genes and detected a moderate number of significant gene pairs (162 pairs) in the gene set.

One of the limitations is that we did not apply scCorr to network analysis, such as weighted gene co-expression  network analysis (WGCNA) and other downstream analyses. Considering that scCorr enables the recovery of gene pair correlations in dropout datasets, it is reasonable to believe that scCorr improves the performance of downstream analysis. Future studies combining scCorr and other existing tools, such as WGCNA, are needed. Compared to imputation methods, one unique feature of graphic-based methods is that scCorr allows users to make analytical decisions based on data visualizations. Easy and fast implementation is another advantage of scCorr. We believe that scCorr can be used independently or as a complementary tool to address dropout effects.

## Conclusions

To address the challenge of dropout in scRNA-seq data analysis, we developed a graph-based method to cluster cells without the need to impute zero values. The scCorr package was tested in two independent datasets from healthy subjects and from a patient with T cell deficiency. Our results show that scCorr is superior to the conventional nonclustering single-cell-based method in detecting correlated gene pairs and improves the efficiency of cell type identification, which is critical for downstream analyses in scRNA-seq data.

## Methods

### Single-cell RNA-seq datasets

#### Dataset 1. Peripheral blood mononuclear cells from healthy subjects

Single-cell data were downloaded from the NCBI Gene Expression Omnibus (GEO) https://www.ncbi.nlm.nih.gov/geo/query/acc.cgi?acc=GSE130228. The dataset was generated using the 10x Genomics platform and contains 15,973 single cells, 21,430 genes and seven cell types. Single-cell data quality control, normalization, and cell type identification were described previously [27].

#### Dataset 2. Ex vivo scRNA-Seq of CD4+ cells in T-bet-deficient and wild-type mice

PBMCs were obtained from healthy controls (wild type) and a patient with inherited human T-bet deficiency [29]. Single-cell RNA sequencing was conducted using the 10X Genomics Chromium Single Cell platform (3′ Reagent kit v2) with Illumina NextSeq 500. Single-cell data were downloaded from the NCBI GEO database (https://www.ncbi.nlm.nih.gov/geo/query/acc.cgi?acc=GSE174804). The dataset contains a total of 18,931 cells and 33,538 genes. Cells with mitochondrial reads greater than > 6% were removed, and 271 duplicated cells were also removed from the analysis. After data normalization and QC, a total of 17,623 cells and 20,849 genes remained.

#### Dataset 3. Transcriptomic and clonal characterization of T cells in the human central nervous system from the single cell expression atlas

Single-cell RNA-seq using 10X Genomics was used to profile T cells from the human cerebrospinal fluid of healthy donors (https://www.ebi.ac.uk/gxa/sc/experiments/E-HCAD-30/results/tsne). In the raw data, a total of 37,586 cells and 22,509 genes were identified. We excluded 2568 cells with high expression of mitochondrial reads, 209 doublets, and 1305 cells with gene number per cell < 400. After QC, 37,377 cells and 19,711 genes remained for analysis.

##### Correlated genes in KEGG pathways

A total of 1532 gene-gene interactions from 347 unique genes were selected from the B cell receptor, p53, and MAPK signaling pathways in the KEGG database (https://www.genome.jp/kegg/pathway.html) (Supplementary Table 1). The 1532 gene pairs served as a reference in the scCorr analysis using the scRNA-seq datasets 1 and 2 described above. In dataset 3, 360 genes and 1781 gene pairs on the same three KEGG pathways were used in the analysis to compare scCorr with three other methods (Supplementary Table 2).

##### Zero value distribution in merged cells

The distribution of the percentage of zeros for each cell (Fig. 1B and D) and each gene (Fig. 1A and C) is shown using histogram plots. Two different gene sets were used: all 21,430 genes and only 347 genes on the 3 KEGG pathways from GSE130228. We examined the zero-value distribution in different sets of merged cells from GSE130228 and from a simulation dataset. We simulated 95% of zero values assuming 23,000 genes and 20,000 cells. We randomly selected two cells and calculated the percentage of zero values in 23,000 genes. Each two-cell selection was performed 1000 times, and the average of the zero percentage in 23,000 was determined. The selection of random cell numbers was repeated until 200 cells were merged. We also simulated different numbers of cells (2000, 8000, and 14,000) and different sets of gene numbers (16,000, 18,000, and 20,000). The distributions of zero values were essentially the same (Supplementary Fig. 1).

##### Zero-value reduction through k-partitioning

The strategy to reduce zero values in gene expression involved using a k-partitioning algorithm to group cells into clusters and calculating the average gene expression in the cluster instead of in single cells. An adjacency distance matrix of single cells was estimated by the single-cell expression profile from the tSNE output and converted into a weighted graph or network using the R package igraph (https://cran.r-project.org/web/packages/igraph/citation.html). The weight values were the length of the edges in the graph. The Louvain algorithm, an efficient graph-clustering method based on the modularity measure and a heuristic approach, was used to group cells into a predefined number of clusters through iteratively splitting and merging cell processes, as shown in the t-SNT plot of cluster numbers 50, 100 and 1000 (Supplementary Fig. 2A). The pseudocodes are included at the end of the Methods section. Cell number distributions from cluster numbers 10–100 and 100–1000 are displayed using box plots.

##### Visualization of cell clusters using the k-partitioning algorithm

Multiple cluster visualization functions were implemented to evaluate the k-partitioning algorithm performance to select desired cluster numbers. In the cluster overlay on the t-SNE plot, each dot represents a cluster with dot size proportional to cluster size (Supplementary Fig. 2B). This provides a convenient function to identify uniformly distributed clusters. Using tree-based visualization of clusters, ladder and circle plots show the evolution of clusters at the different cluster numbers (Supplementary Fig. 3). Dot size is proportional to the cell number in one cluster, and lines between dots track the cluster development. Supplementary Fig. 3A presents a tree of 20–40 clusters from top to bottom. The tree can be arranged in a circular shape suitable for displaying a large number of clusters (Supplementary Fig. 3B and C); inner and outer circles correspond to the top and bottom trees, respectively.

Similarly, the results of k-partitioning clusters in the Dataset 3 are presented in Supplementary Fig. 4. tSNE plot shows relatively homogenous cells in the dataset. scCorr enables the partition of 10, 40, 100, and 400 mini clusters.

##### Gene-gene correlation analysis

Pearson and Spearman correlation coefficients at the single-cell and cluster levels were calculated between gene pairs extracted from KEGG pathways, as exemplified in the scatter and violin plots of *MARPK1-DUSP2* (Supplementary Fig. 5). The correlation *p* values were estimated using generalized linear models (glm). At the cluster level, the average gene expression of cells in a cluster was used as the gene expression value. The top 10 correlated gene pairs by *p* values based on 100 clusters were selected to evaluate the effect of the number of clusters on the correlation. A total of 16 cluster sets were used: 40–100 and 100–1000 in increments of 10 and 100, respectively. The relationships of glm *p* values and Pearson and Spearman correlation coefficients vs. different numbers of clusters are illustrated using a line plot (Supplementary Fig. 6). In the dataset 3, scCorr identified 626 significant pairs of correlated genes among 1769 pairs, while the uncluttering method identified 612 significant pairs of correlated genes among 1781 pairs. Correlation The correlation coefficients of paired genes by scCorr arewere greater than those of gene pairs by non-clusteringthe nonclustering method, while the *p* values of gene pairs by scCorr arewere smaller than those of gene pairs by non-clustering the nonclustering method (Supplementary Fig. 7). Thus, scCoOrr outperforms non-clusteringthe nonclustering individual cell gene-gene correlation method even in relatively homogenous cells.

##### Time estimation for k-partitioning clusters

We implemented calculation time for different numbers of cell clusters. In our analysis, using Rtsne with perplexity = 30 and max_iter = 2000, the x and y coordinate regions were approximately from − 50 to 50. Before performing graph-based clustering, we suggest that the x and y coordinate regions are scaled from − 200 to 200 or − 400 to 400 in cell numbers 5967 and 15,973, respectively. Supplementary Fig. 8 shows estimated times for a range of scaled clusters. On a tSNE plot scale of − 50 to 50 of 5976 cells, when the scale range is 200, the running time is smallest, regardless of the number of clusters. In a plot of 15,973 cells, a scale range of 400 appears to be the most rapid option, regardless of the number of clusters. The different colors for the lines represent the numbers of clusters.

### Codes

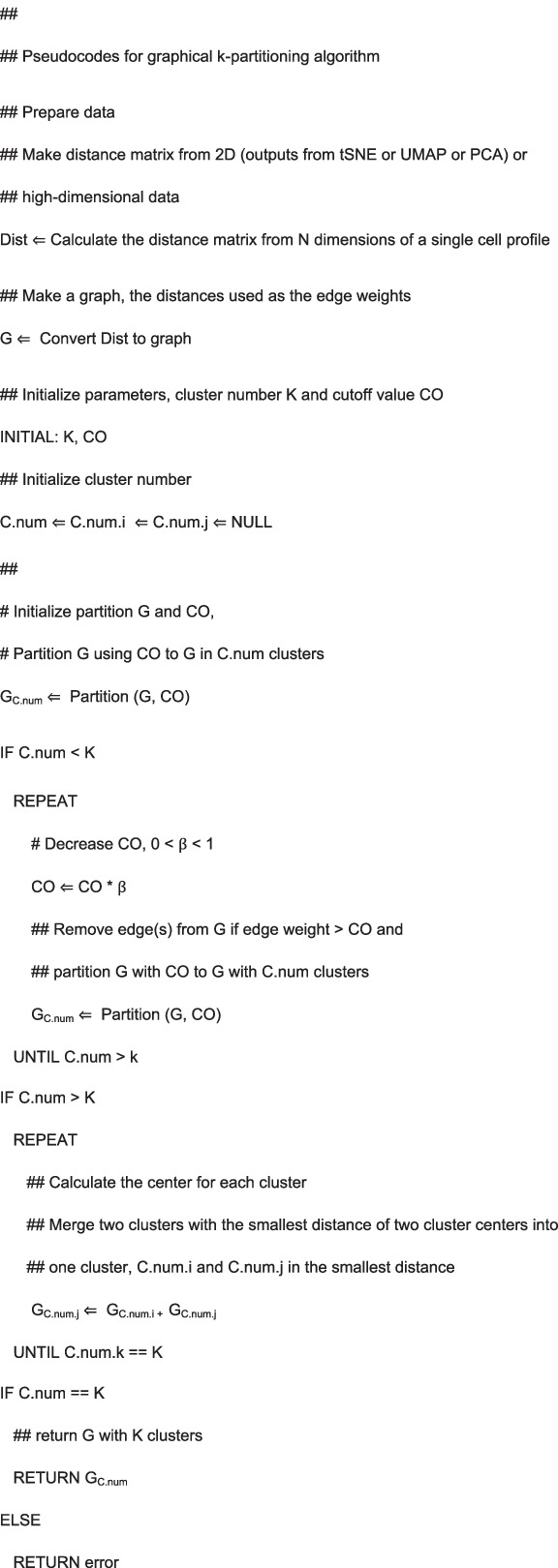



## Supplementary Information

**Additional file 1.**

**Additional file 2.**

**Additional file 3.**

## Data Availability

Sequencing data have been deposited in GEO under the accession numbers GSE130228 (https://www.ncbi.nlm.nih.gov/geo/query/acc.cgi?acc=GSE130228) and GSE174804 https://www.ncbi.nlm.nih.gov/geo/query/acc.cgi?acc=GSE174804). Both datasets are open to the public. The third dataset is available at https://www.ebi.ac.uk/gxa/sc/experiments/E-HCAD-30/results/tsne. Code availability is publicly available from GitHub under the CBIIT-CGBB (https://github.com/CBIIT-CGBB/scCorr).

## Abbreviations

*ATF4*: Cyclic AMP-dependent transcription factor ATF-4
AUC: Area under the curve
DeepImpute: Deep neural network Imputation
*DUSP2*: Dual specificity protein phosphatase 2
KEGG: Kyoto Encyclopedia of Genes and Genomes
*KLF13*: Krueppel-like factor 13
*LASP1*: LIM and SH3 domain protein 1
*MAN2A1*: Alpha-mannosidase 2
*MAP 7D1*: MAP 7 domain-containing protein 1
*MAPK1*: Mitogen-activated protein kinase 1
*MAPKAPK2*: MAP kinase-activated protein kinase 2
PBMC: Peripheral blood mononuclear cell
scRNA-seq: Single-cell RNA-sequencing
scCorr: Single-cell gene-gene correlation
tSNE: t-distributed stochastic neighbor embedding
WGCNA: Weighted Gene Co-expression Network Analysis

## References

[1] Haque A, Engel J, Teichmann SA, Lonnberg T (2017). A practical guide to single-cell RNA-sequencing for biomedical research and clinical applications. Genome Med.

[2] Paik DT, Cho S, Tian L, Chang HY, Wu JC (2020). Single-cell RNA sequencing in cardiovascular development, disease and medicine. Nat Rev Cardiol.

[3] Keren-Shaul H, Spinrad A, Weiner A, Matcovitch-Natan O, Dvir-Szternfeld R, Ulland TK, David E, Baruch K, Lara-Astaiso D, Toth B (2017). A unique microglia type associated with restricting development of Alzheimer's disease. Cell.

[4] Haghverdi L, Buttner M, Wolf FA, Buettner F, Theis FJ (2016). Diffusion pseudotime robustly reconstructs lineage branching. Nat Methods.

[5] Yao C, Sun HW, Lacey NE, Ji Y, Moseman EA, Shih HY, Heuston EF, Kirby M, Anderson S, Cheng J (2019). Single-cell RNA-seq reveals TOX as a key regulator of CD8(+) T cell persistence in chronic infection. Nat Immunol.

[6] Gladka MM, Molenaar B, de Ruiter H, van der Elst S, Tsui H, Versteeg D, Lacraz GPA, Huibers MMH, van Oudenaarden A, van Rooij E (2018). Single-cell sequencing of the healthy and diseased heart reveals cytoskeleton-associated protein 4 as a new modulator of fibroblasts activation. Circulation.

[7] Chen GM, Chen C, Das RK, Gao P, Chen CH, Bandyopadhyay S, et al. Integrative bulk and single-cell profiling of pre-manufacture T-cell populations reveals factors mediating long-term persistence of CAR T-cell therapy. Cancer Discov. 2021;11(9):2186-99. 10.1158/2159-8290.CD-20-1677. Epub 2021 Apr 5. PMC8419030 33820778

[8] Buettner F, Natarajan KN, Casale FP, Proserpio V, Scialdone A, Theis FJ, Teichmann SA, Marioni JC, Stegle O (2015). Computational analysis of cell-to-cell heterogeneity in single-cell RNA-sequencing data reveals hidden subpopulations of cells. Nat Biotechnol.

[9] Kharchenko PV, Silberstein L, Scadden DT (2014). Bayesian approach to single-cell differential expression analysis. Nat Methods.

[10] Vallejos CA, Risso D, Scialdone A, Dudoit S, Marioni JC (2017). Normalizing single-cell RNA sequencing data: challenges and opportunities. Nat Methods.

[11] Kim JK, Kolodziejczyk AA, Ilicic T, Teichmann SA, Marioni JC (2015). Characterizing noise structure in single-cell RNA-seq distinguishes genuine from technical stochastic allelic expression. Nat Commun.

[12] Arisdakessian C, Poirion O, Yunits B, Zhu X, Garmire LX (2019). DeepImpute: an accurate, fast, and scalable deep neural network method to impute single-cell RNA-seq data. Genome Biol.

[13] Liu J, Liu X, Ren X (2019). Li G: scRNAss: a single-cell RNA-seq assembler via imputing dropouts and combing junctions. Bioinformatics.

[14] Tracy S, Yuan GC, Dries R (2019). RESCUE: imputing dropout events in single-cell RNA-sequencing data. BMC Bioinformatics.

[15] Lu T, Park S, Zhu J, Wang Y, Zhan X, Wang X, Wang L, Zhu H, Wang T (2021). Overcoming expressional drop-outs in lineage reconstruction from single-cell RNA-sequencing data. Cell Rep.

[16] Ran D, Zhang S, Lytal N, An L (2020). scDoc: correcting drop-out events in single-cell RNA-seq data. Bioinformatics.

[17] Eraslan G, Simon LM, Mircea M, Mueller NS, Theis FJ (2019). Single-cell RNA-seq denoising using a deep count autoencoder. Nat Commun.

[18] Kobak D, Berens P (2019). The art of using t-SNE for single-cell transcriptomics. Nat Commun.

[19] Talwar D, Mongia A, Sengupta D, Majumdar A (2018). AutoImpute: autoencoder based imputation of single-cell RNA-seq data. Sci Rep.

[20] van Dijk D, Sharma R, Nainys J, Yim K, Kathail P, Carr AJ, Burdziak C, Moon KR, Chaffer CL, Pattabiraman D (2018). Recovering gene interactions from single-cell data using data diffusion. Cell.

[21] Qiu P (2020). Embracing the dropouts in single-cell RNA-seq analysis. Nat Commun.

[22] Peng T, Zhu Q, Yin P, Tan K (2019). SCRABBLE: single-cell RNA-seq imputation constrained by bulk RNA-seq data. Genome Biol.

[23] Qi Y, Guo Y, Jiao H, Shang X (2020). A flexible network-based imputing-and-fusing approach towards the identification of cell types from single-cell RNA-seq data. BMC Bioinformatics.

[24] Li WV, Li JJ (2018). An accurate and robust imputation method scImpute for single-cell RNA-seq data. Nat Commun.

[25] Bageritz J, Willnow P, Valentini E, Leible S, Boutros M, Teleman AA (2019). Gene expression atlas of a developing tissue by single cell expression correlation analysis. Nat Methods.

[26] Lun AT, McCarthy DJ, Marioni JC (2016). A step-by-step workflow for low-level analysis of single-cell RNA-seq data with Bioconductor. F1000Res.

[27] Hu Y, Ranganathan M, Shu C, Liang X, Ganesh S, Osafo-Addo A, Yan C, Zhang X, Aouizerat BE, Krystal JH (2020). Single-cell transcriptome mapping identifies common and cell-type specific genes affected by acute Delta9-tetrahydrocannabinol in humans. Sci Rep.

[28] Blondel VD, Guillaume JL, Lambiotte R, Lefebvre E. Fast unfolding of communities in large networks; 2008. arXiv:0803.0476v2 [physics.soc-ph

[29] Yang R, Yang R, Weisshaar M, Mele F, Benhsaien I, Dorgham K, et al. High Th2 cytokine levels and upper airway inflammation in human inherited T-bet deficiency. J Exp Med. 2021;218(8). 10.1084/jem.20202726 PMC8225679 34160550

[30] Pappalardo JL, Zhang L, Pecsok MK, Perlman K, Zografou C, Raddassi K, et al. Transcriptomic and clonal characterization of T cells in the human central nervous system. Sci Immunol. 2020;5(51). 10.1126/sciimmunol.abb8786 PMC8567322 32948672

