# Supplementary material for: A novel graph-based k-partitioning approach improves the detection of gene-gene correlations by single-cell RNA sequencing
Source: BMC Genomics. 2022 Jan 7;23:35. doi: 10.1186/s12864-021-08235-4 (PMC8740455; doi:10.1186/s12864-021-08235-4)
Supplement: Supplementary file 2 — Additional file 2. [file 12864_2021_8235_MOESM2_ESM.docx]

**A novel graph-based k-partitioning approach improves the detection of gene-gene correlations by single-cell RNA sequencing**

Heng Xu^1*^, Ying Hu ^2*^, Xinyu Zhang ^3,4^, Bradley E. Aouizerat ^5^, Chunhua Yan ^2^, Ke Xu ^3,4^

1. Department of Psychiatry, Perelman School of Medicine, University of Pennsylvania, Philadelphia, PA, USA.

2. Center for Biomedical Information and Information Technology, National Cancer Institute, MD, USA.

3. Department of Psychiatry, Yale School of Medicine, New Haven, CT, USA.

4. Connecticut Veteran Healthcare System, West Haven, CT, USA.

5. Bluestone Center for Clinical Research, College of Dentistry, New York University, New York, NY, USA

*** contributed equally**

**Supplementary Figures**

**Supp Figure 1**. Dropout effect in simulated dataset. Distributions of zero values of gene expression in four sets of simulated datasets (**A**) and in the scRNA-seq dataset with 21,430 genes and 15,973 peripheral blood mononuclear cells (**B**).

**Supp Figure 2**. t-SNE plot-based k-partitioning cluster. (**A**). All cells are clustered as the size of 50, 100, and 1,000 groups. (**B**). Three identical structure plots show a proportion of a cluster size. Each dot represents a cluster, and the size of the dot is proportional to the cluster size.

**Supp Figure 3**. Tree-based visualization of cell clusters by the k-partitioning algorithm. Ladder clusters, N=20-40 (**A**); Circle clusters, N=20-40 (**B**); and Circle clusters, N=100-1,000 (**C**). The size of each dot represents the proportion of the cell number in one cluster. A line connects two closest adjacent clusters.

**Supp Figure 4**. Correlation of a co-expression gene pair: DUSP2 and MAPK1 estimated by the non-clustering singal cell method (**A**) and by scCorr clustering method (**B**).

**Supp Figure 5**. t-SNE plot-based k-partitioning clusters in the dataset 3.

**Supp Figure 6**. Comparisons between scCorr and nonclustering method: Coexpressed gene pairs detected by scCorr and by nonclustering single cell in the dataset 3.

**Supp Figure 7**. Correlation of top 10 co-expressed gene pairs in different numbers of partitioned clusters in CD4+ T cells: evaluated by p values and r values using Person Correlation and Spearman Correlation.

**Supp Figure 8**. Estimation of computation time for tSNE-based k- partitioning cluster. xy.coordinate represents the regions of scaling. (A) Estimation of xy coordinates (B) In a tSNE plot scale of -50 to 50 of 5,976 cells, the running time is the shortest at the scale range is 200 regardless of the number of clusters. (C) In a plot of 15,973 cells, a scale range of 400 appears most rapid regardless of the number of clusters. Different color of line represents the numbers of clusters.
